# Choice of numerical implementation of spatial contrast calculation impacts microcirculation quantitation in laser speckle contrast imaging

**DOI:** 10.1117/1.JBO.30.4.046006

**Published:** 2025-04-16

**Authors:** Marc Chammas, Frédéric Pain

**Affiliations:** Université Paris-Saclay, Institut d’Optique Graduate School, CNRS, Laboratoire Charles Fabry, Palaiseau, France

**Keywords:** laser speckle contrast imaging, spatial contrast, 2D filters, convolution, blood flow

## Abstract

**Significance:**

Laser speckle contrast imaging (LSCI) allows noninvasive imaging of microcirculation. Its scope of clinical applications is growing, yet the literature lacks a comparison of the accuracy of methods used to compute the spatial contrast $K_{s}$ from which the blood flow index is derived.

**Aim:**

We aim to evaluate the impact on flow quantitation of different computational approaches used to derive $K_{s}$.

**Approach:**

We compare numerical calculation of $K_{s}$ in Python and ImageJ applied to noise-free simulated data and to experimental data acquired *in vivo* in anesthetized mice. The estimation of the decorrelation time $\tau_{c}$, inversely proportional to the blood flow index, is carried out following two approaches: LSCI asymptotic estimation and fitting the multiple exposure speckle imaging (MESI) model to $K_{s}(T)$.

**Results:**

For simulation data, we found variations of up to 58% for the blood flow index in the LSCI approach. Nonlinear fitting of the MESI model was less affected with discrepancies of only a few percent. Considering experimental data, the LSCI approximation led to $K_{s}$ with relative differences (up to 35%) depending on the calculation methods. The noise and limited exposure time strongly limited the accuracy of the LSCI asymptotic estimation. Adjustment of the MESI model to the data led to consistent values of $\tau_{c}$ in the 0.05 to 1 ms range with significant variations depending on the method used to calculate $K_{s}$.

**Conclusions:**

Numerical methods used to calculate $K_{s}$ should be precisely acknowledged and validated against direct calculation to ensure accuracy. *Uniform* filter approach leads to accurate $K_{s}$ values and is 100 times more computationally efficient than the Direct calculation. Other investigated methods lead to various levels of errors in flow index estimation using LSCI. Errors are minimized using larger kernels. MESI derivation of $\tau_{c}$ is not immune but less affected by such methodological biases.

## 1 Introduction

Laser speckle contrast imaging (LSCI) relies on the illumination of a sample with coherent light from a laser. Speckles are interferometric patterns that are observed when coherent light is used to illuminate objects. When a sample contains moving scatterers such as red blood cells in a blood vessel, the pattern evolves over time. Recording these time-varying speckle patterns with a camera for a given exposure time leads to a local blur in the image. The local statistics of the resulting grainy speckle pattern contain information on the movements of the scatterers within the sample. The most common metric used is the speckle contrast, defined as the local ratio of the standard deviation to the mean of the gray levels of the pixels $K_{S}=\sigma /\langle I\rangle$. It can be computed in spatial, temporal, or spatio-temporal ensembles of neighboring pixels.^1^ For computing efficiency, the images are processed numerically using sliding convolution filters. In most implementations, the spatial speckle contrast is calculated over windows of 5×5 or 7×7  pixels. This allows a reasonable spatial resolution and adequate statistical accuracy.^2^ The speckle contrast values quantify the blur of the local speckle pattern, which is in turn related to the local decorrelation time $\tau_{c}$ and ultimately to the speed of the scatterers movements.^3^ As the exact relationship between $\tau_{c}$ and the speed is not known, a relative metric is used, the blood flow index (BFI), estimated as $1/\tau_{c}$. In LSCI, the BFI can be derived as $\mathrm{BFI}=1/{\mathrm{TK}}^{2}$ if $T/\tau_{c}>100$.^4^ Multiple exposure speckle imaging (MESI) is an improved variant of LSCI, where several values of $K_{s}$ are obtained at different exposure times. A mathematical model can then be fitted to the resulting $K_{s}(T)$ providing a more robust estimation of the local decorrelation time $\tau_{c}$.^5^ The relative blood flow changes are then estimated through the relative changes of the inverse correlation time (relative ICT) defined as $1/\tau_{c}$.^6^ Regarding quantitation and comparison of measurements between systems, several instrumental and methodological parameters have been pointed out and investigated throughout the years, including the effect of the speckle to pixel size ratio, the contribution of specular reflected light, and the amount of system noises.^1^^,^^7^ Although the accuracy of the metrics detailed above depends on the accuracy of $K_{s}$ calculation, the effect of the numerical methods implemented to calculate the speckle contrast was less investigated. A computationally efficient approach was proposed based on the calculation of the variance as the mean of squared local intensities values minus the square of the mean of these values.^8^ However, the actual numerical implementation of $K_{s}$ calculation is seldom provided in the literature. Studying the accuracy of $K_{s}$ numerical calculations and its potential impact on BFI or ICT is relevant in light of the current efforts toward improved standardization and relative and absolute quantitation.^7^^,^^9^^–^^12^ The aim of this work is to compare the accuracy of different numerical approaches for spatial contrast calculations and the corresponding $\tau_{c}$ evaluations. The computational speed and accuracy in both LSCI and MESI implementations for imaging of microcirculation have been investigated on simulated and experimental raw speckle data.

## 2 Methods

### 2.1 Speckle Images Datasets

#### 2.1.1 Simulated data

Dataset #1 is composed of simulated images of time-integrated speckle patterns at 15 different exposure times. The simulated camera exposure times are 0.05, 0.1, 0.2, 0.5, 0.8, 1, 2, 3.5, 5, 10, 15, 20, 30, 40, and 60 ms. These exposure times span two orders of magnitude allowing to sample the decrease of the speckle contrast with increasing exposure time in MESI studies of mice brain microcirculation.^13^ Images have a bit depth of 12 bits and dimensions of 1000×1000  pixels. The simulations were carried out following the implementation described by James et al.^14^ The method allows the simulation of time-integrated speckle patterns for custom adjustable decorrelation functions and parameters. The geometry is a homogeneous, purely diffusive media with no static scatterers. A decorrelation function corresponding to multiple photon scattering in ordered flow was simulated. Other flow and scattering regimes can be simulated, but we chose specifically this one as *in vivo* measurements of $g_{1}(\tau_{c})$ have shown that it is relevant for arterioles in functional areas of the mice brain.^15^ Two sets of data with decorrelation times of 0.1 and 1 ms were simulated in the range of $\tau_{c}$ values that were observed experimentally in mice brain arterioles.^15^ A ratio of 3 pixels per speckle was set in the simulation to ensure speckle grain sampling was better than the Shannon–Nyquist criteria.^2^

#### 2.1.2 Experimental *in vivo* data

Dataset #2 is composed of experimental raw MESI speckle images acquired *in vivo* at the surface of exposed cortical tissues of a mouse. These data have been obtained previously.^16^ In this previous study, the number of exposure times was limited to 6 with a maximum of 20 ms to reduce the duration of an MESI sequence and improve the overall temporal resolution of the technique. *In vivo* imaging was carried out in accordance with the European Directive 2010/63/UE (Ethical project #CEB-03-2018) regarding the care and use of laboratory animals. *In vivo* recordings were carried out in 4-month-old, anesthetized mice (male, C57BL6, mean weight = 48 g) after a craniotomy was performed over the barrel cortex. Anesthesia was carried out with a cocktail of ketamine (100  mg/kg Imalgen) and medetomidine (0.5  mg/kg), injected intraperitoneally. The anesthesia level was adjusted as necessary throughout the experiment. Surgery begins when the mouse no longer responds to the hindpaw pinch. During the entire experiment, the animal was placed on a thermostatically controlled heating pad. Body temperature was maintained rectally at 37°C. After the animal was secured in a stereotactic frame, imaging was carried out over regions of $∼3\times 2\;{\mathrm{mm}}^{2}$ over the barrel cortex. A low-temperature agarose layer (1.2%) and a microscope coverslip were placed over the dura matter. A stabilized laser diode (SGL, Shanghai, China, 634 nm 200 mW laser diode) in free space was modulated in time and intensity using an acousto-optic modulator (AOM) (AA Opto-Electronic, Orsay, France) driven by an arbitrary waveform generator (Keysight 336001 Series, Penang, Malaysia). The AOM allows modulating the first diffraction order of the signal. A fixed aperture diaphragm was used to select the first order (modulated) of diffraction while filtering the non-diffracted light as well as eventual higher diffraction orders. After 15 min of laser warm-up, light was shone on the exposed cortical tissue at an oblique incidence to minimize specular reflection contributions. Cycling of the modulated laser pulses is synchronized with image acquisition. The camera was a CMOS Orca Flash 2.8 (Hamamatsu Photonics, Massy, France) with a frame rate of 46 fps at full frame. It was c-mounted on a MZ16 microscope (Leica Microsystems, Nanterre, France) providing a × 3.3 magnification resulting in a field of view of $∼3\times 2\;{\mathrm{mm}}^{2}$. A ratio of 2.3 pixels per speckle grain was measured experimentally. Dataset #2 is composed of 774 frames cycling through 6 exposure times (1, 2, 5, 10, 15, and 20 ms) corresponding to 129 MESI sequences.

### 2.2 Numerical Implementations of Speckle Contrast Calculations

The Python and ImageJ implementations of $K_{s}$ calculations are summarized in Table 1. Python was chosen as it is open source and has become one of the common languages for teaching and research in the biomedical engineering community. Versions of software and Python modules are summarized in Table 2. Data were processed using a MacBook Air with an M1 microchip and 16 GB of random access memory (RAM). The reference calculation of $K_{s}$ is referred to as the *Direct* method. It was implemented using nested “for” loops based on the mathematical definitions of the mean and variance. Other methods rely on vectorized approaches using the “sums algorithm”^8^ and the calculation of local squared, summed, and averaged values using convolution filters. The corresponding Python and ImageJ scripts are available in a Zenodo repository.^17^ For both ImageJ macro and *Scipy uniform_filter* Python function, the data are processed as 3D stacks, which is convenient for LSCI and MESI data consisting of stacks (i.e., movies) of 2D image frames. On the contrary, *Scipy correlated2d* (adapted from Ref. 18) and *SimpleITK* convolution methods require 2D arrays as inputs, which implies the use of “for” loops to process 3D stacks corresponding to frame sequences. By default, ImageJ uses circular convolution kernels, whereas *Uniform_filter* and *Scipy correlated2d* use square kernels. *SimpleITK* convolution is implemented with 2D circular kernels that are defined in a custom separate function. Square kernels have a side dimension of 5 or 7 pixels. For circular kernels, radii of 2 or 3 pixels are set, resulting in kernels of 5 or 7 pixel diameter. The default edge management for the different methods is self-explanatory and named “reflect,” “constant padding,” and “zero padding” *for Uniform_filter*, *Correlated2d*, and *SimpleITK* convolutions, respectively. They can be customized to some extent for *Uniform_filter* and *Correlated2d* to “mirror” or “symmetrical” values around the edges. The edge management affects only the values on the sides of the images. Consequently, to avoid eventual bias, we have cropped the borders of the final speckle contrast images to remove the affected edge pixels. For each dataset, the corresponding $K_{s}$ images are calculated using the different methods listed in Table 1. For dataset #1, as the imaging field is homogeneous, the resulting mean $K_{s}$ is calculated as the average of all pixels for each contrast frame. For dataset #2, the spatial contrast frames are calculated for all 774 frames, and then, for each exposure time, the corresponding 129 frames are averaged, providing an average MESI sequence with six exposure times. Then, three regions of interest (ROIs) are drawn, over two arterioles and a parenchyma region [see Fig. 2(a)]. Care was taken not to include the vessel walls in the arterial ROIs. The speckle contrast values within each ROI are averaged to provide the mean $K_{s}$ as a function of the exposure times.

**Table 1:** Numerical methods implemented to calculate $K_{s}$.

Name	Language	Libraries/modules	Input	Parameters
*Uniform_filter*	Python	Scipy.ndimage	Images stack	2D or 3D kernel
*Correlated2d*	Python	Scipy.signal	Single 2D image	2D kernel
*SimpleITK*	Python	SimpleITK	Single 2D image	2D circular kernel
*ImageJ_Macro*	FIJI	Built-in Filters	Images stack	2D circular kernel

**Table 2:** Versions of software and Python modules.

Python	Scipy	Numpy	SimpleITK	ImageJ
3.10.14	1.14.0	2.0.0	2.3.1	2.14.0 1.54f

### 2.3 Evaluation of Decorrelation Time

BFI is defined as $1/\tau_{c}$. For LSCI data, it is estimated as $\mathrm{BFI}=1/{\mathrm{TK}}^{2}$, following the asymptotic approximation.^4^ For MESI, $\tau_{c}$ values are estimated using a nonlinear regression of the MESI model on the data^5^^,^^19^

$$K(T,\tau_{c})={\left\{\beta \rho^{2}(\frac{e^{-2x}-1+2x}{(2x{)}^{2}})+4\beta \rho (1-\rho )(\frac{e^{-x}-1+x}{x^{2}})+v_{ne}+v_{\text{noise}}\right\}}^{\frac{1}{2}},$$ (1)

T is the exposure time, β is a constant depending on instrumental parameters, ρ is the proportion of dynamic scatterers involved in the speckle patterns, x is $T/\tau_{c}$, $v_{ne}$ accounts for nonergodic contribution, and $v_{\text{noise}}$ accounts for noise contributions. To fit the MESI model to the dataset #1, the parameter ρ was set to 1, $v_{\mathrm{noise}}$ and $v_{ne}$ were set to 0 corresponding to the simulation’s “ideal” conditions, i.e., an imaging field composed only of dynamic scatterers, and no experimental noises. For experimental dataset #2, the nonergodic and experimental noises’ contributions were pooled in a single parameter $v_{\text{noise}}$. The parameters were constrained within the following physical boundaries: 0.05<β<0.5, 0.1<ρ<1, $0<\tau_{c}<10$, $0<v_{\text{noise}}<0.30$.

## 3 Results and Discussion

The local spatial speckle contrast for all frames in datasets #1 was computed using the numerical methods from Table 1. It corresponds to the time requested to calculate the contrast images for one MESI sequence of 1000×1000  pixels images with 15 different exposure times. We have first evaluated the computation times for each method and then the influence on the contrast values, resulting $\tau_{c}$ in LSCI and MESI.

### 3.1 Computing Time

Execution times were evaluated in Python using the time module. ImageJ macro was timed in batch mode (i.e., no screen output occurred during the calculation). For each Python approach listed in Table 1, an average timing was evaluated based on 100 iterations of the same calculation to smooth out system overheads. The absolute execution times are only indicative as they highly depend on the hardware used for calculation. The relative execution times are more relevant. The results in Table 3 indicate that the direct estimation is ∼100 to 1000 times slower than the other methods. Overall, *simpleITK* and *Uniform_filter* are the fastest. *Correlated2d* implementation is 8 to 15 times slower depending on the kernel dimension. *ImageJ* timing is twice that of *uniform*. Methods were applied using kernels of 5 or 7 pixel side or radius. Except for *Scipy_correlated2D* and *SimpleITK*, the execution times of which were almost doubled, the other timings were not significantly affected by the kernel dimensions.

**Table 3:** Execution times for different implementations of speckle calculation on dataset #1 ($\tau_{c}=0.1\;\mathrm{ms}$).

Kernel dimension (pixels)	Execution times (s)				
	*Direct*	*Uniform_filter*	*Correlated2d*	*SimpleITK*	*ImageJ*
5	410	0.2	1.6	0.2	0.4
7	407	0.2	3.0	0.4	0.4

### 3.2 Speckle Contrast Values for Simulated Data

**Fig. 1:**
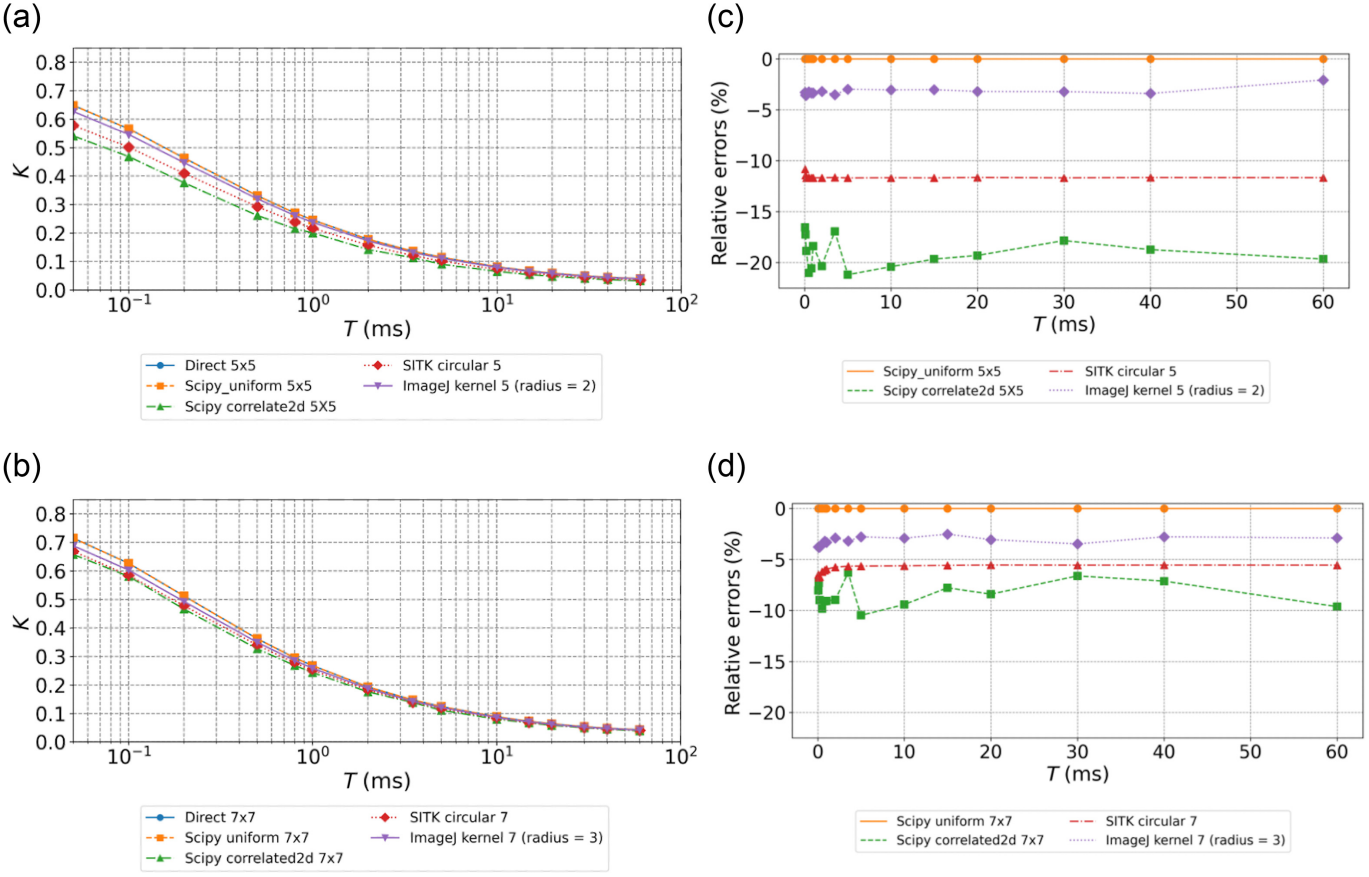
Ks(T) derived for the different methods and relative errors for simulated data at $\tau_{c}=0.1\;\mathrm{ms}$. *Direct* calculation is considered the reference. (a) Ks(T) for kernels of 5×5; (b) Ks(T) for kernels of 7×7; (c) relative errors (kernels of 5×5); and (d) relative errors (kernels of 7×7).

All the different methods were applied to calculate the mean contrast for each frame of dataset #1. The resulting $K_{s}$ for kernels with a 5 or 7 pixel side or diameter are shown in Figs. 1(a) and 1(b) for the data with a simulated decorrelation time $\tau_{c}=0.1\;\mathrm{ms}$. Considering the *Direct* calculation as the reference, the relative errors for each method are presented in Figs. 1(c) and 1(d). The uniform method provides values that are almost identical to those of the *Direct* calculation. *ImageJ* calculation also presents values with less than 5% difference with the reference. *SimpleITK* and finally *Correlated2d* give the values with the strongest discrepancies, up to 20% for *Correlated2d* with a 5 pixel window. As expected, the calculations using larger kernels lead to lower discrepancies as the statistics of the speckle patterns are better sampled.^2^ Similar trends are observed for data with a simulated decorrelation time $\tau_{c}=1\;\mathrm{ms}$ (Fig. S1 in the Supplementary Material).

### 3.3 Estimation of the Decorrelation Times for Simulated Data

Table 4 shows the estimations of $\tau_{c}$ derived from the LSCI asymptotic approximation and from the nonlinear regression of the MESI model on $K_{s}(T)$ derived using all methods applied to dataset #1. For each exposure time, values of $K_{s}$ are calculated as the average of all pixels. In the LSCI asymptotic approximation, regardless of the method used to compute $K_{s}$, the estimated $\tau_{c}$ is closer to the expected simulated value when $T/\tau_{c}$ increases. As expected, the LSCI estimations for the shorter exposure times are poor in all cases. LSCI estimations at the longest exposure time (60 ms) are fair for the data simulated with $\tau_{c}=0.1\;\mathrm{ms}$, but poor for the data simulated with $\tau_{c}=1\;\mathrm{ms}$. Comparing the methods, for LSCI, the lowest accuracy is observed for *Correlated2d* in line with the discrepancies observed for $K_{s}$ calculation [Figs. 1(c) and 1(d)]. On the contrary, $\tau_{c}$ values derived from nonlinear regressions of the MESI model are less affected by the numerical method used. Considering the analysis with 7×7 kernels, all the MESI estimates of $\tau_{c}$ are within a few percentages from the expected value. The values of $\tau_{c}$ derived from the $K_{s}$ calculated with the 5×5 kernels are less accurate, with up to 17% discrepancy for the simulated data at $\tau_{c}=1\;\mathrm{ms}$ analyzed with *Scipy correlated2d*. As the ICT is computed as the inverse of $\tau_{c}$, it is biased by the same relative error as $\tau_{c}$. Regarding the kernel shape, most speckle imaging studies use square kernels as they are easy to implement in array processing. However, square kernels can introduce directional bias, whereas round kernels treat data isotropically. Nevertheless, the results show that the kernel shape is less critical than the numerical method and the dimension of the kernel used. Despite the use of kernels with different shapes, *Uniform* (square kernel) and *ImageJ* (round kernel) provide the best results considering the *Direct* method as the reference. All methods have better accuracy when larger kernels are used, regardless of the shape of the kernel.

**Table 4:** Estimated values of the decorrelation time $\tau_{c}$ (in ms) for dataset #1 obtained using the LSCI asymptotic approximation or by fitting the MESI model to the $K_{s}(T)$ derived using each numerical method and kernels of side or diameter of 5 and 7 pixels.

Asymptotic approximation	*Direct*	*Uniform_filter*	*Correlated2d*	*SimpleITK*	*ImageJ*
Simulated $\tau_{c}=0.1\;\mathrm{ms}$, kernel side or diameter of 5 pixels					
Texp=1 ms	0.060	0.060	0.040	0.047	0.056
Texp=20 ms	0.069	0.069	0.045	0.054	0.065
Texp=40 ms	0.079	0.079	0.052	0.062	0.074
Texp=60 ms	0.095	0.095	0.061	0,074	0.091
MESI regression	0.106	0.106	0.091	0.101	0.105
Simulated $\tau_{c}=0.1\;\mathrm{ms}$, kernel side or diameter of 7 pixels					
Texp=1 ms	0.072	0.072	0.059	0.063	0.067
Texp=20 ms	0.082	0.082	0.069	0.073	0.077
Texp=40 ms	0.093	0.093	0.081	0.083	0.088
Texp=60 ms	0.112	0.112	0.092	0.100	0.106
MESI regression	0.105	0.105	0.101	0.105	0.106
Simulated $\tau_{c}=1\;\mathrm{ms}$. kernel side or diameter of 5 pixels					
Texp=1 ms	0.321	0.321	0.228	0.252	0.299
Texp=20 ms	0.626	0.626	0.407	0.489	0.585
Texp=40 ms	0.646	0.646	0.417	0.504	0.605
Texp=60 ms	0.655	0.655	0.425	0.511	0.612
MESI regression	0.935	0.935	0.838	0.858	0.918
Simulated $\tau_{c}=1\;\mathrm{ms}$, kernel side or diameter of 7 pixels					
Texp=1 ms	0.394	0.394	0.333	0.342	0.364
Texp=20 ms	0.743	0.743	0.618	0.660	0.699
Texp=40 ms	0.765	0.765	0.636	0.680	0.718
Texp=60 ms	0.774	0.774	0.675	0.689	0.726
MESI regression	0.976	0.976	0.995	0.944	0.995

### 3.4 Limitations of Simulation Data

Simulations from dataset #1 do not account for experimental noises that may affect significantly the speckle contrast values. These include camera noise (dark, read-out, fixed noise pattern, and shot noise) as well as potential instability of the illumination, contamination with noncoherent ambient light, or unwanted movements (vibrations or respiratory movements). Most of these contributions can be significantly reduced through careful design of the experimental set-up. However, they cannot be completely avoided. In addition, simulations in dataset #1 represent a purely dynamic, homogeneous media, whereas *in vivo* data are a mix of dynamic and static contributions. In Sec. 3.5, we have further evaluated the impact of the choice of the numerical implementation of $K_{s}$ calculation for real data acquired *in vivo* in mice.

### 3.5 Speckle Contrast Calculation and Decorrelation Times Estimation on *In Vivo* Data

**Fig. 2:**
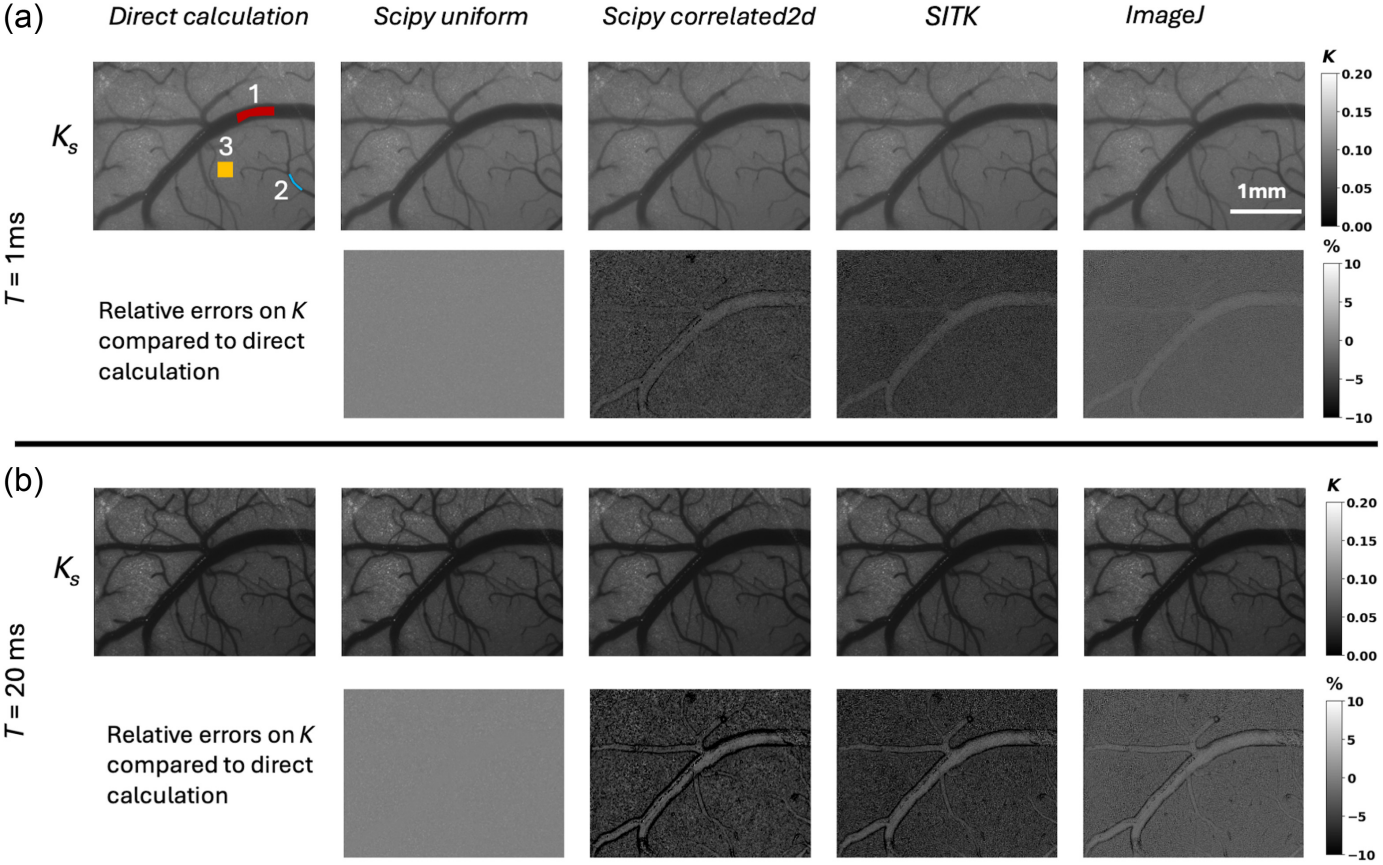
(a) $K_{s}$ maps for all methods (kernel side or diameter of 7 pixels) at 1 ms exposure time, averaged over 129 MESI sequences (top row) and relative error maps compared with the direct method (bottom row). Three regions of interest are drawn over a large arteriole (ROI#1), a small arteriole (ROI#2) and parenchyma (ROI#3); (b) same as (a) for 20 ms exposure time.

**Fig. 3:**
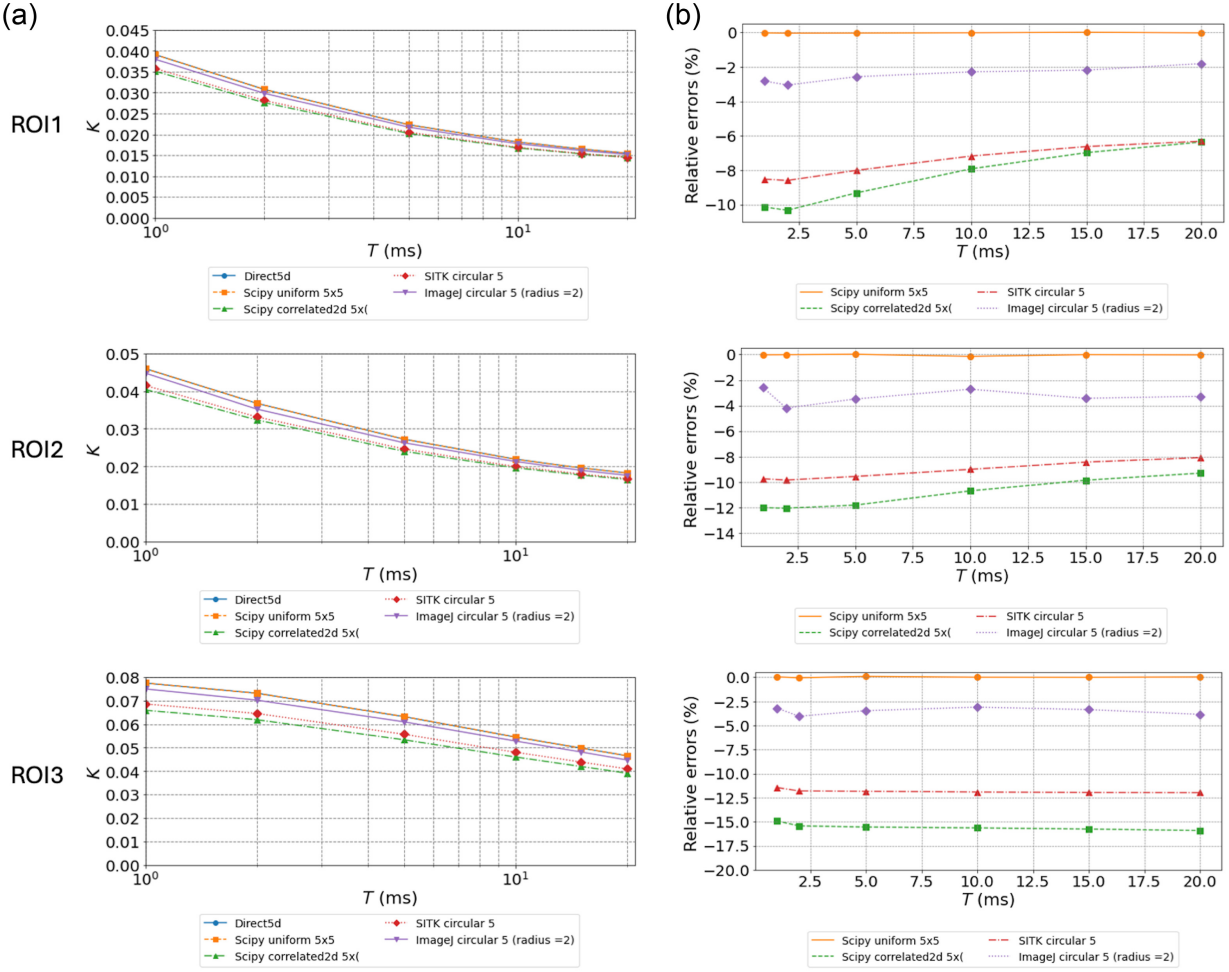
Evaluation of $K_{s}$ and relative errors for dataset #2 using all methods and kernels of side or diameter of 5 pixels. (a) $K_{s}$ values as a function of exposure times for all methods and kernels of 5 pixel side or diameter. (b) Relative errors for the $K_{s}$ values relative to the *Direct* method as a function of exposure times for all methods. Each row corresponds to one of the ROIs drawn in Fig. 2(a).

**Fig. 4:**
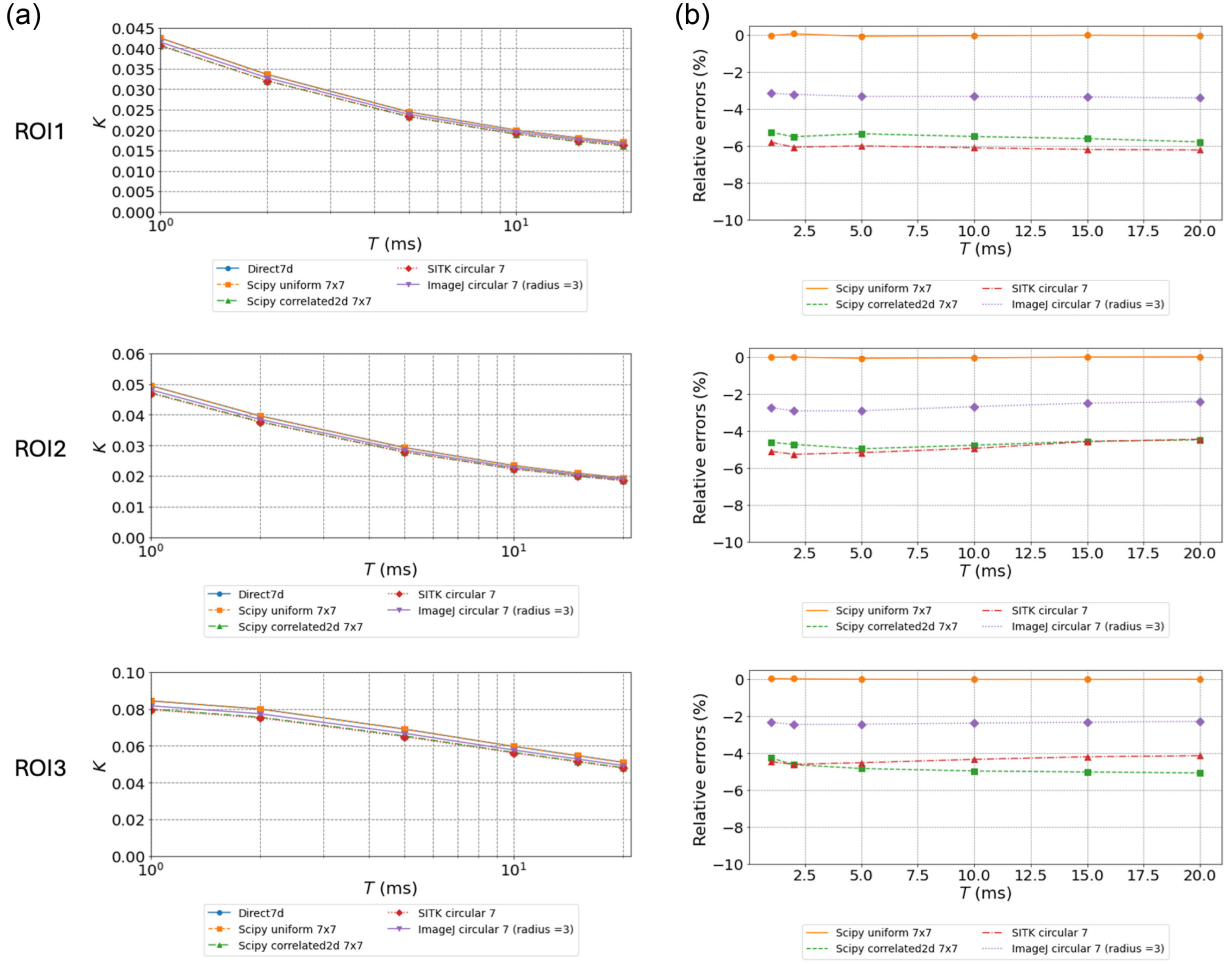
Evaluation of $K_{s}$ and relative errors for dataset #2 using all methods and kernels of side or diameter of 7 pixels. (a) $K_{s}$ values as a function of exposure times for all methods and kernels of 7 pixel side or diameter. (b) Relative errors for the $K_{s}$ values relative to the *direct* method as a function of exposure times for all methods. Each row corresponds to one of the ROIs drawn in Fig. 2(a).

Speckle contrast images derived from real data present inherently a nonhomogeneous field of view. Figure 2(a) shows, for all numerical methods, the speckle contrast images obtained for an exposure time of 1 ms and the corresponding relative differences with the contrast $K_{s}$ images obtained with the *Direct* method. Figure 2(b) shows the speckle contrast images and corresponding relative error maps for an exposure time of 20 ms. Although $K_{s}$ images look very similar for all methods, the relative error images show significant local differences in $K_{s}$ compared with the *Direct* calculation. Here, for the purpose of comparison, the same scale was applied to the relative error images. Figures S2 and S3 in the Supplementary Material show the $K_{s}$ and relative error images at all exposure times for all methods. The largest relative errors are observed for the *Correlated2D* (−25%) and *SimpleITK* (−20%) methods similar to what was observed for simulated data. *Uniform* methods provide almost error-free $K_{s}$ images, whereas *ImageJ* leads to intermediate errors in the ±5% range. The relative errors are relatively homogeneous inside the larger vessels, the smaller vessels, and parenchyma. For larger vessels, the gradients of integrated speckle values lead to larger errors in the wall regions, as observed in Fig. 2(b), particularly for *Correlated2d*. This bias is more pronounced at longer exposure times and can also be observed for *SimpleITK* and *ImageJ* for an exposure time of 20 ms. For all methods, the mean contrast $K_{s}(T)$ was calculated over the three ROIs, as shown in Fig. 2(a). The results are plotted in Figs. 3 and 4 as a function of exposure time for the different calculation methods and kernels of 5×5 and 7×7  pixels, respectively.

The absolute values of contrasts are much lower in the experimental data compared with the simulated dataset due to fast decorrelation and added experimental noises. Consistent with expectations, the lowest values are observed in the region with presumably the fastest flow (large arteriole, ROI1), and the highest $K_{s}$ are observed in the region with the slowest flow (ROI3). The small arteriole in ROI2 shows intermediate values. For kernels of 5 pixel side or diameter, the ranking and trends in terms of relative accuracy on $K_{s}(T)$ calculations are similar to those observed for calculations on simulated data [compare Figs. 1(a), 1(b), and 3]. The values of $K_{s}$ obtained with *Uniform* are almost identical to those obtained with the *Direct* calculation. Regarding other methods, *ImageJ* analysis remains close to the reference value followed by *SimpleITK* convolution and *Correlated2d*. For these two implementations, the relative errors are much smaller compared with those obtained on simulated data. This could be attributed to the averaging of 129 MESI sequences for the *in vivo* dataset, whereas simulated data are single frames for each exposure time. In addition, the errors decrease slightly with exposure times for these methods. Indeed, the relative errors observed for *Scipycorrelated2d* are twice higher for ROI3 (parenchyma) compared with those for the large arteriole region (ROI1). This follows the values of the $K_{s}$ in these two regions, which are about twice higher in ROI3 than in ROI1. Regarding the influence of the kernel dimensions, as observed for the simulated data, using larger kernels leads to significantly lower errors (compare Figs. 3 and 4) notably for *Scipycorrelated2d* and *SimpleITK*. This can again be attributed to improved estimations of speckle pattern statistics. For 7×7 kernels and for all methods, the relative errors remain constant over the different exposures [Fig. 4(a)]. In the LSCI asymptotic approximation, $\tau_{c}$ is estimated directly from $1/K_{s}^{2}$, so in a first-order approximation, the relative uncertainty on $\tau_{c}$ is twice that on $K_{s}$. Considering the values of relative errors on $K_{s}$ for the maximum exposure time of 20 ms in Fig. 3, this theoretically leads to deviations on $\tau_{c}$ between 5% and 35% for kernels of 5×5  pixels. For kernels of 7×7  pixels, the deviations are limited to 5% to 12%. Finally, the graphs of $K_{s}(T)$ in Figs. 3 and 4 show that the maximal exposure time considered in this dataset does not allow reaching a clear asymptotic value of $K_{s}$, thus preventing an accurate estimation of $\tau_{c}$ using the LSCI approximation.

Table 5 presents the values of $\tau_{c}$ derived from the MESI model for all methods and mean $K_{s}(T)$ from the three ROIs, as shown in Fig. 2(a). The values obtained for kernels of 7 and 5 pixel side or diameter are shown. The values of $\tau_{c}$ estimated for both kernel dimensions show similar trends, but the $\tau_{c}$ values are systematically lower for kernels of 5 pixel side or diameter. Considering $K_{s}(T)$ obtained by the *Direct* and *Uniform_filter* methods using kernels of 7×7, the values of $\tau_{c}$ derived from the MESI model are very close and range between 0.499 ms for the parenchyma (ROI3) region down to 0.135 ms for the small arteriole (ROI2) and 0.094 ms in the largest arteriole (ROI1), in line with what was quantified *in vivo* in arterioles in the mouse brain using direct decorrelation function measurement.^15^ Compared with these values, $\tau_{c}$ values derived from $K_{s}$ calculated using *Correlated2d*, *SimpleITK*, and *ImageJ* are systematically underestimated for all ROIs. Underestimations of $\tau_{c}$ are similar for *SimpleITK* and *Scipycorrelated2d* and significantly lower for *ImageJ*.

**Table 5:** Estimated values of the decorrelation time $\tau_{c}$ (in ms) obtained for each ROI in Fig. 2(a) by fitting the MESI model on $K_{s}(T)$ calculated with all the different numerical implementations with kernel side or diameter of 5 or 7 pixels.

*ROI*	*Arteriole diameter*	*Direct*	*Uniform_filter*	*SimpleITK*	*Correlated2D*	*ImageJ*
Kernel side or diameter of 5 pixels						
ROI1	178±10 μm	0.076	0.076	0.061	0.064	0.074
ROI2	38±10 μm	0.115	0.115	0.085	0.09	0.108
ROI3	Parenchyma	0.342	0.342	0.204	0.228	0.299
Kernel side or diameter of 7 pixels						
ROI1	178±10 μm	0.094	0.094	0.083	0.082	0.087
ROI2	38±10 μm	0.135	0.135	0.123	0.122	0.129
ROI3	Parenchyma	0.499	0.501	0.388	0.379	0.429

## 4 Conclusions

The study shows the strong impact on $\tau_{c}$ blood flow index quantitation of the numerical methods used to calculate the spatial contrast. The observed significant underestimations of $K_{s}$ can be partly minimized if larger kernels dimensions are used and if contrast frames’ averaging is carried out. Among the methods tested, the *Uniform_filter* with a 2D square filter gave results almost identical to the Direct estimation but for a calculation time several orders of magnitude shorter. *ImageJ* circular filter implementation also provided fair results, within a few percentages of the *Direct* calculation. Other implementations (*Scipy correlated2d*, *SimpleTK*) gave much larger errors’ event for large kernels and substantial contrast frames’ averaging, which should be avoided. For the experimental data, the presence of noises and the lower values of contrast lead to a limited accuracy of the asymptotic LSCI approximation at a single exposure time. For all implementations, MESI analysis leads to physiologically consistent flow index estimation with $\tau_{c}$ decreasing with the increasing diameter of arterioles (associated to faster flow) with $\tau_{c}$ values in the same order of magnitude as absolute quantitation obtained in mice brain.^15^ The MESI model is fitted to the experimental curves of $K_{s}$ as a function of time, the shape of which is preserved by the different algorithms. This minimizes the impact of $K_{s}$ underestimations by the numerical methods. However, *ImageJ*, *SimpleITK*, and *Correlated2D* methods all led to significant underestimations of $\tau_{c}$. In conclusion, although the choice of the software implementation for $K_{s}$ calculation may seem trivial; the present data show that this often overlooked point definitely plays a role in the blood flow index quantitation in LSCI and MESI imaging. The Python method based on *Scipy Uniform* filter provides accurate calculations of $K_{s}$ calculations and subsequent estimation of $\tau_{c}$. It was also one of the fastest in terms of computation efficiency. In light of the continuous theoretical and experimental efforts and recent advances toward better relative and even absolute quantitation of blood flow with laser speckle imaging,^7^^,^^9^^,^^11^^,^^12^^,^^20^^,^^21^ we believe that the numerical implementation used in LSCI or MESI to calculate the speckle contrast should be systematically compared against the *Direct* calculation to avoid numerical bias.

## Supplementary Material

10.1117/1.JBO.30.4.046006.s01

## Data Availability

The data and code supporting this study are available in the Zenodo open repository at https://doi.org/10.5281/zenodo.14178966.
