# Supplementary material for: Choice of numerical implementation of spatial contrast calculation impacts microcirculation quantitation in laser speckle contrast imaging
Source: J Biomed Opt. 2025 Apr 16;30(4):046006. doi: 10.1117/1.JBO.30.4.046006 (PMC12003051; doi:10.1117/1.JBO.30.4.046006)
Supplement: Supplementary file 1 [file JBO_030_046006_SD001.pdf]

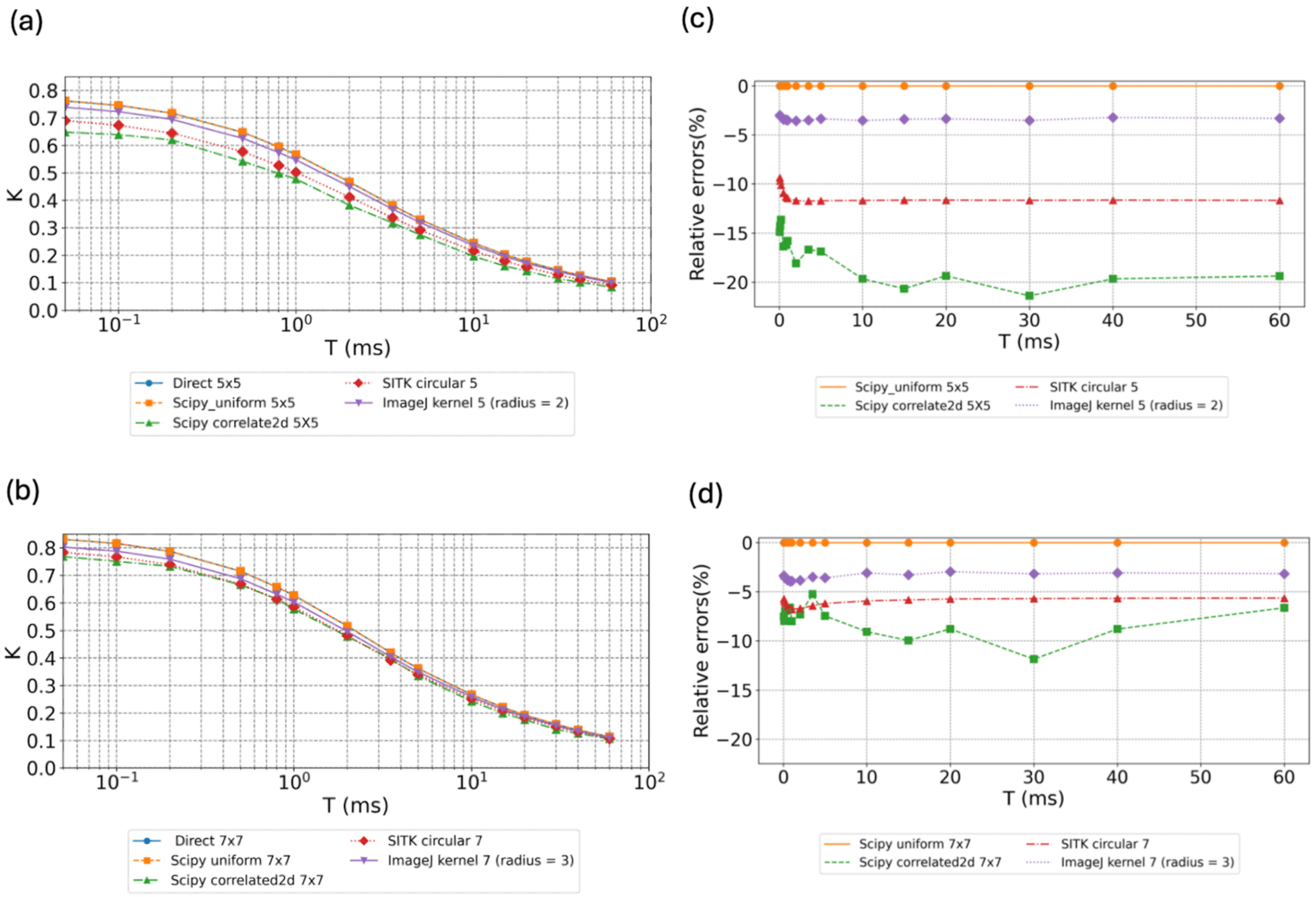

Supplementary Fig.S1 $K_s(T)$ derived for the different methods and relative errors for simulated data at τ_c = 1 ms. Direct calculation is considered as the reference. (a) $K_s(T)$ for kernels of 5 x 5 (b) $K_s(T)$ for kernels of 7 x 7. (c) Relative errors (kernels of 5 x 5) and (d) Relative errors (kernels of 7 x 7).

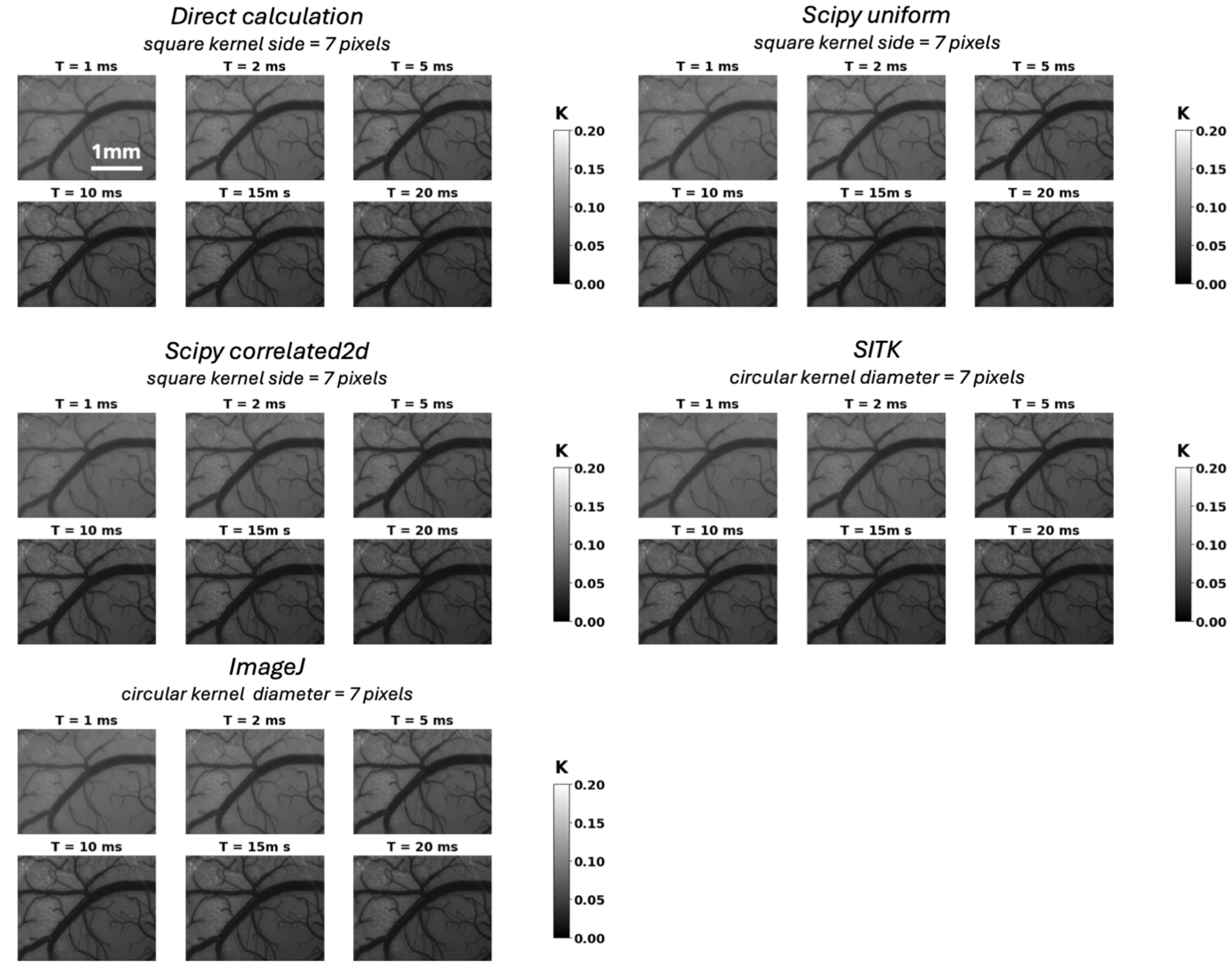

Supplementary Fig.S2 Images of $K_s(T)$ for dataset #2 derived by the Direct, Uniform, SITK, Correlated2d and *ImageJ* calculation methods with a kernel of 7 pixels side or diameter.

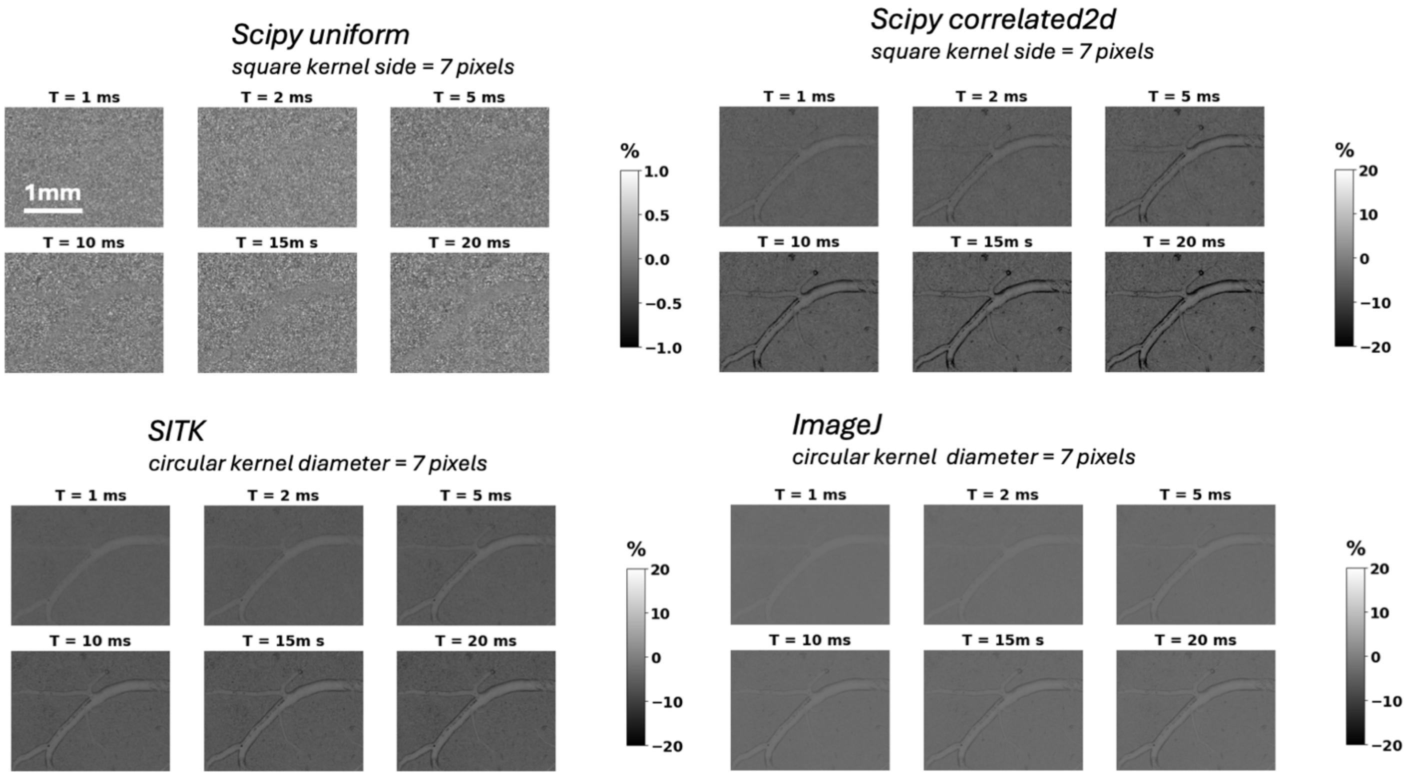

Supplementary Fig.S3 Images of relative errors (reference images are those obtained by *Direct* calculation shown on Fig.S2 for *Ks(T)* calculated on dataset #2 by the *Uniform, SITK, Correlated2d* and *ImageJ* methods with a kernel of 7 pixels side or diameter.
